# Traffic flow prediction based on spatiotemporal encoder-decoder model

**DOI:** 10.1371/journal.pone.0321858

**Published:** 2025-05-30

**Authors:** Yuanming Ding, Wei Zhao, Lin Song, Chen Jiang, Yunrui Tao

**Affiliations:** Communication and Network Key Laboratory, Dalian University, Dalian, China; National University of Defense Technology, CHINA

## Abstract

To more effectively capture the periodic and dynamic changes in urban traffic flow and the spatiotemporal correlation of complex road networks, a new traffic flow prediction method, the Enhanced Spatiotemporal Graph Convolutional Network Encoder-Decoder Model (ESGCN-EDM), is proposed. The model achieves a significant enhancement in prediction accuracy through the introduction of the attention-based Personalized-enhanced Fusion Graph Convolutional Network (aPFGCN) and the Temporal Convolutional Bidirectional Long Short-Term Memory (TCBiL) module. The aPFGCN module effectively reduces the dimensionality of features and decreases model complexity to obtain the final node feature representation by personalizing the adjustment of node influence coefficients and applying Fourier transform and inverse transform techniques. Additionally, by incorporating an attention mechanism, it enhances the model’s ability to focus on important information and effectively captures the spatial topological relationships within the traffic network. The TCBiL module integrates 1D convolution with BiLSTM to form a unified temporal feature extraction module. The 1D convolution is utilized to extract local features from the time series, while the BiLSTM captures long-term dependencies within the time series. This allows for simultaneous feature extraction and temporal modeling, thereby enhancing the model’s efficiency and performance, and strengthening its ability to model time series. In the encoder part of the ESGCN-EDM, the aPFGCN is combined with the TCBiL to handle the spatiotemporal coupling interactions of the road network. The decoder part then performs multi-step predictions based on spatiotemporal sequences using the TCBiL and CNN, generating high-dimensional representations. Extensive experiments conducted on two real-world road traffic datasets demonstrate that the ESGCN-EDM model consistently outperforms other benchmark models in 1-hour, 30-minute, and 15-minute traffic flow predictions. Specifically, on the PeMSD8 dataset, the model achieves reductions in MAE, RMSE, and SMAPE by 7.9%, 2.1%, and 16.9%, respectively, compared to the AMRGCN model for 1-hour predictions. Similarly, on the PeMSD4 dataset, the model reduces MAE, RMSE, and MAPE by 1.8%, 1.1%, and 3.0%, respectively. These results validate the efficacy of the proposed model and its ability to significantly enhance the accuracy of traffic flow forecasting.

## Introduction

With the rapid pace of urbanization, urban traffic pressure continues to escalate, and traffic congestion emerges as a pivotal issue impeding urban growth and development. Traffic congestion not only diminishes the efficiency of urban operations but also gives rise to a series of social issues, including environmental pollution and energy waste [1]. According to the World Bank, economic losses attributable to traffic congestion in major global cities can reach 1%–3% of GDP [2]. As urban traffic systems become increasingly complex, there is an urgent need for innovative solutions to address this formidable urban challenge [3]. The establishment of Intelligent Transportation Systems (ITS) plays a crucial role in alleviating traffic pressure. Traffic flow prediction, as a core component of ITS, can forecast future traffic conditions using historical data. Therefore, the development of models that can accurately predict traffic flow is of great significance for optimizing route planning and enhancing traffic management [4].

The technology of traffic flow prediction has undergone significant evolution over time. The development of traffic flow prediction techniques progresses from traditional statistical methods to machine learning, and subsequently to deep learning. Early research primarily employed methods based on statistical theory, such as the Historical Average (HA) [5], Autoregressive Integrated Moving Average (ARIMA) [6], and Kalman Filtering [7]. These methods are widely used due to their computational simplicity, especially Kalman Filtering, which effectively handled time-series prediction problems in linear systems through dynamic system modeling and state estimation. However, these methods are limited in their ability to capture nonlinear characteristics, resulting in constrained prediction accuracy. As the complexity of traffic flow data became better understood, researchers began to turn to machine learning methods, such as K-Nearest Neighbors (KNN) [8] and Support Vector Machines (SVM) [9]. These methods, which optimize parameters through adaptive learning, significantly improved prediction accuracy. For instance, Priambodo *et al*. [10] proposed a spatiotemporal K-NN traffic state prediction method based on statistical features of adjacent roads, which improved traffic flow prediction accuracy by identifying highly correlated roads within neighboring areas. Mladenović *et al*. [11] introduced a supervised machine learning approach to predict the total and average monthly nighttime traffic volumes on Serbian state roads. Their study found that models based on KNN algorithms and regression trees performed best, enabling accurate predictions of total and average monthly nighttime traffic volumes at selected traffic counting locations for the following year. Toan *et al*. [12] developed a method utilizing SVM and KNN to enhance short-term traffic flow prediction accuracy and model training efficiency. Experiments demonstrated that this method outperformed other baseline approaches in rolling predictions, and increasing the rolling prediction horizon was shown to further improve prediction accuracy. However, these traditional methods have limitations when applied to modern urban transportation networks. They require manual feature selection and struggle with handling complex spatial structures and dynamic changes. Additionally, they fail to capture the long-term dependencies and intricate spatiotemporal interactions of traffic flow.

In recent years, deep learning techniques have achieved significant advancements in traffic flow prediction. Long Short-Term Memory networks (LSTM) [13], known for their exceptional ability to model temporal sequences, have become the mainstream approach in traffic flow prediction. Leveraging their strengths in handling complex problems and automatically extracting spatiotemporal features from data, LSTM-based methods have emerged as state-of-the-art technologies in the field of traffic flow prediction. Researchers continue to explore how to better utilize deep learning to uncover higher-order temporal dependencies in traffic data, aiming to achieve more efficient and accurate traffic flow predictions. For instance, the stacked LSTM model proposed by Li *et al*. [14] enhances the fitting capability for nonlinear functions by increasing the depth of the neural network, thereby improving prediction accuracy. Guo [15] combines LSTM with variational autoencoder techniques to handle historical and missing data, further strengthening the capability of temporal sequence modeling. Given that traffic flow data are typical spatiotemporal sequence data, although LSTM-based methods have improved prediction accuracy, research solely based on traditional LSTM still faces limitations in extracting dynamic features of time series and integrating external influencing factors. Moreover, these studies often fail to fully consider spatial correlations. The variation characteristics of traffic flow in different regions may be influenced by factors such as geographic location, urban functions, and transportation infrastructure. Traffic flow exhibits complex temporal patterns, including periodicity (e.g., morning and evening peaks, weekend patterns) and trends (e.g., long-term growth or decline), while also being susceptible to disruptions from unexpected events (e.g., accidents, weather conditions). Spatially, traffic flow demonstrates intricate interdependencies. For instance, congestion on one road segment may impact adjacent segments or even propagate to more distant areas. Such spatial dependencies are often nonlinear and influenced by the topology of the road network. Additionally, traffic data may contain substantial noise (e.g., sensor malfunctions, data transmission errors), which can adversely affect model training and prediction. Consequently, traditional LSTM-based methods are unable to effectively identify and leverage these features, making it difficult to fully uncover the intrinsic patterns of traffic flow dynamics.

To overcome the aforementioned limitations, researchers have begun exploring hybrid models that combine Convolutional Neural Networks (CNN) with LSTM to separately extract temporal and spatial features from traffic flow data. Zhang *et al*. [16] employed Complementary Ensemble Empirical Mode Decomposition (CEEMD) for signal decomposition to reduce the impact of noise on traffic flow data prediction. They utilized CNN and LSTM to fully exploit the spatiotemporal features of the data, thereby enhancing the accuracy of model predictions and improving the learning efficiency of neural networks. Yang *et al*. [17] propose a hybrid deep learning approach based on CNN and LSTM for traffic flow prediction. This method employs CNN to extract spatial features of traffic flow and uses LSTM to capture temporal sequence variations, achieving accurate predictions of traffic flow conditions. The study found that simulation data from small-scale traffic systems can be used to train large-scale systems, thanks to the scale invariance of statistical mechanics models. Experimental results indicated that this method performed exceptionally well in terms of prediction accuracy, significantly improving the precision of predictions. Xia *et al*. [18] introduced a hybrid deep learning method called RSAB-ConvGRU, based on Residual Self-Attention and Bidirectional GRU, for traffic flow prediction. This method uses the Conv-GRU module to extract spatiotemporal features and combines residual self-attention mechanisms to enhance the model’s expressive power. Additionally, the bidirectional GRU module captures the periodic characteristics of traffic data. Experiments demonstrated that the model effectively handles the nonlinearity and uncertainty of traffic data, significantly improving prediction accuracy. However, CNNs are only capable of processing Euclidean data, which are characterized by well-defined distances and directions, with relationships between data points measurable by Euclidean distance [19]. In real-world traffic networks, it is evident from the layout of sensors and network nodes that traffic flow data exhibit a typical non-Euclidean structure. The relationships between data points in such structures are complex and cannot be measured by simple Euclidean distances [20]. Traffic networks can be viewed as graphs, where nodes represent intersections or sensors, and edges represent roads. The relationships between nodes in traffic flow data depend not only on physical distances but are also influenced by road topology, traffic regulations, and real-time traffic conditions. As traffic conditions change over time, the relationships between nodes also evolve.

To extract spatiotemporal features from traffic flow data and more effectively handle its non-Euclidean structural properties, Graph Convolutional Networks (GCNs) have been introduced into the field of traffic flow prediction by incorporating graph structure information. GCNs are not dependent on the specific scale or shape of the road network, allowing them to be flexibly applied to traffic networks of varying scales and types. Whether it is a large-scale urban road network or a small-scale local area, GCNs can adapt to different application scenarios by adjusting the graph structure and model parameters. This strong generalization capability makes GCNs highly promising for a wide range of traffic flow prediction tasks. The ASTGCN model proposed by Guo *et al*. [21] integrates spatiotemporal attention mechanisms with GCNs, achieving precise traffic flow prediction. Song *et al*. [22] develop the STSGCN model, which effectively captures and models complex local spatiotemporal correlations through its spatiotemporal synchronization mechanism. Xia *et al*. [23] proposed a short-term traffic flow prediction model that integrates community detection, federated learning, and GCN to mitigate the time-consuming training, high communication costs, and data privacy risks associated with increasing data volume in global GCN frameworks, thereby achieving accurate and secure short-term traffic flow prediction. Xing *et al*. [24] propose a smart city traffic flow prediction method based on graph convolution, LSTM networks, and reinforcement learning (RL-GCN). This method processes urban traffic network data features using graph convolution, learns temporal information through LSTM networks, and combines reinforcement learning algorithms to formulate optimal traffic control strategies, significantly improving the accuracy of urban traffic flow prediction. Huang *et al*. [25] introduced the MD-GCN model, which effectively extracts spatiotemporal correlations in traffic flow data by combining multi-scale temporal convolution and dual graph convolutional networks, leading to a notable improvement in traffic flow prediction accuracy. However, due to the extensive parameter calculations and data processing involved in feature extraction, the computational complexity of the model remains high. Although many excellent research achievements have been made, the realization of high-accuracy traffic flow prediction has not yet been fully resolved [26], necessitating continuous exploration and innovation in this field. Specifically, existing methods still face limitations in handling complex spatiotemporal dependencies and nonlinear traffic flow variations, such as difficulties in fully capturing dynamic correlations among nodes in traffic networks or effectively modeling multi-scale temporal features in long time series.

In response to the aforementioned challenges, this paper proposes a novel deep learning-based traffic flow prediction method—the Enhanced Spatiotemporal Graph Convolutional Network Encoder-Decoder Model (ESGCN-EDM). This model effectively extracts spatiotemporal correlations in traffic flow prediction sequences by introducing an attention-based Personalized-enhanced Fusion Graph Convolutional Network (aPFGCN) and a Temporal Convolutional Bidirectional Long Short-Term Memory (TCBiL) module, thereby improving the model’s efficiency and performance while significantly enhancing prediction accuracy.

(1) The aPFGCN module first adjusts the influence coefficients of GCN nodes in a personalized manner, then performs feature extraction in the spectral domain through Fourier transform, effectively reducing feature dimensionality and model complexity. Subsequently, it remaps the fused features back to the time domain using inverse Fourier transform to obtain the final node feature representations. Finally, by incorporating an attention mechanism, the module enhances the model’s ability to focus on critical information, effectively capturing the spatial topological relationships within the traffic network.

(2) The TCBiL module integrates 1D convolution with Bidirectional Long Short-Term Memory (BiLSTM) to form a unified temporal feature extraction module. The 1D convolution is employed to extract local features from time series, while BiLSTM is utilized to capture long-term dependencies within the time series. This integration enables simultaneous feature extraction and temporal modeling, improving the model’s efficiency and performance while enhancing its capability to model time series.

(3) In the encoder part of ESGCN-EDM, aPFGCN and TCBiL are combined to handle the spatiotemporal coupling interactions of the road network. The decoder part, on the other hand, employs TCBiL and CNN to perform multi-step predictions based on spatiotemporal sequences, generating high-dimensional representations that accurately capture the interdependencies between historical and predicted traffic flows. Extensive experiments on two real-world traffic flow datasets, PeMSD4 and PeMSD8, demonstrate that the proposed ESGCN-EDM outperforms other comparative models in 1-hour, 30-minute, and 15-minute predictions, validating the model’s effectiveness. Furthermore, ablation experiments are conducted to analyze the contributions of each module to the model’s performance, confirming the critical roles of the aPFGCN and TCBiL modules in enhancing prediction accuracy.

## Problem description and related concepts

### Traffic flow prediction tasks

Traffic flow prediction, a complex spatiotemporal issue, requires forecasting future traffic volumes based on historical data and the structure of the road network. This prediction is crucial for alleviating urban traffic congestion, promoting environmental sustainability, and ensuring public safety [27].

In this study, the traffic road network is modeled as an undirected graph G=(V,E,A), where *V* denotes the set of nodes, representing traffic sensors, and |V|=N denotes the number of sensors; The set *E* contains the connections between sensors, namely the set of edges. $𝒜\in {ℛ}^{N*N}$ serves as an adjacency matrix of the traffic road network, mapping the connectivity of the entire traffic network. The matrix *A* consists solely of elements 0 and 1. If two sensors are directly adjacent on the same road, the corresponding matrix element is 1; otherwise, it is 0. The traffic flow observed on *G* at time *t* is represented by the graph signal $X_{t}\in {ℛ}^{N*F}$, where *F* is feature dimensions of each node. The historical traffic flow data at time *t* can be defined as $X=(X_{t-H+1},...,X_{t-1},X_{t})\in {ℛ}^{H*N*F}$, with *H* being the length of the historical observations data. Denote the predicted traffic flow as $Y=(X_{t+1},...,X_{t+P})\in {ℛ}^{P*N*F}$, where *P* is the length of the predicted data [28].

The objective of traffic flow prediction [29] is to construct a predictive model φ that, based on the given historical traffic data *X* and the corresponding road network structure *G* at the given time, forecasts the traffic flow *Y* at each node over the future *T*_*p*_ time period. In other words, the model achieves the mapping φ from (X;G) to *Y*, expressed as φ:(X,G)→Y.

### Graph convolutional network

Traditional traffic flow prediction methods [30] [31] often partition the transportation network into grids during prediction to leverage the network structure for capturing spatial features. However, such approaches frequently overlook the topological connectivity of the transportation network [32]. GCNs are a deep learning framework specifically designed to analyze data structured in the form of graphs. By aggregating information from neighbors, GCNs can effectively process graph-structured data, making them a powerful tool for extracting complex spatial-topological relationships in transportation networks [28]. Although more advanced graph convolution methods exist, such as Graph Attention Networks (GAT) [33] and GraphSAGE [34], the simplicity and effectiveness of GCNs make them the preferred choice for this study.

Consider a batch of graph data with *N* nodes, where the corresponding node features are represented by an N×D feature matrix *X*. The relationships between nodes are represented by an adjacency matrix *A* of the same size. Both *A* and *X* serve as inputs to the model [35]. As a neural network layer, the GCN aggregates and transforms node features based on the structure defined by *A*, propagating information to capture spatial dependencies. The feature propagation mechanism is as follows:

$$H^{\left(l+1\right)}=\sigma ({\tilde{D}}^{-1/2}\tilde{A}{\tilde{D}}^{-1/2}H^{\left(l\right)}W^{\left(l\right)})$$ (1)

where $\tilde{A}=A\;+\;I$, *I* is the identity matrix, $\tilde{D}$ is the degree matrix of $\tilde{A}$, with ${\tilde{D}}_{i,j}=\sum_{j}A_{i,j}$. *H*(*l*) and *W*(*l*) represent the output features of layer (*l*–1) and the weight matrix of the layer *l*, respectively. σ denotes a nonlinear activation function. In the GCN, the feature matrix is first multiplied by the adjacency matrix to gather node features. These features are then scaled through interaction with the parameter matrix *W*(*l*), and finally, the aggregated node feature matrix is obtained by applying the activation function.

### Encoder-decoder architecture

**Fig 1:**
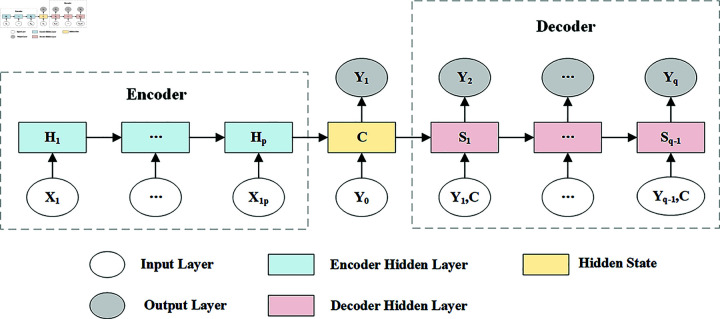
Encoder-decoder architecture.

The encoder-decoder architecture is a popular model structure for seq-to-seq tasks in deep learning. After initial great success in machine translation tasks, this architecture has been extended to a variety of application scenarios including natural language processing, automatic generation of image descriptions, speech recognition, and traffic flow prediction [36]. This architecture, as shown in Fig 1, enables efficient learning and generation of the target sequence by decomposing the complex sequence transformation task into encoding and decoding stages.

The encoder plays a crucial role in encoding historical data into a compact hidden state, denoted as *C*. It captures the essential features and patterns of the input sequence. The decoder, on the other hand, utilizes the information encapsulated in *C* to predict future data. Since the decoder relies on the predictions from the previous time step and the encoded hidden state as inputs for subsequent time steps, this architecture offers high flexibility. The encoder-decoder structure can effectively handle situations where the lengths of the input and output sequences differ. It allows the encoder and decoder to adopt different types of architectures [37], as long as they satisfy the following mapping:

$$X=[X_{1},...,X_{p}]\overset{seq2seq}{\to }Y=[Y_{1},...,Y_{q}]$$ (2)

Where *p* and *q* represent the sequence lengths processed by the encoder and decoder, respectively, which can differ. In traffic flow prediction tasks, the length of historical traffic flow data (input sequence) often differs from the number of future time steps (output sequence) to be predicted. For example, historical data from the past 12 time steps might be used to predict traffic flow for the next 3, 6, or 12 time steps. The encoder-decoder architecture can adapt well to such variations by encoding historical sequences of different lengths into a fixed-length context vector through the encoder. The decoder then generates a prediction sequence of the corresponding length based on this context vector. Therefore, the encoder-decoder structure is particularly well-suited for traffic prediction tasks that require multi-step forecasting [27].

## Models

**Fig 2:**
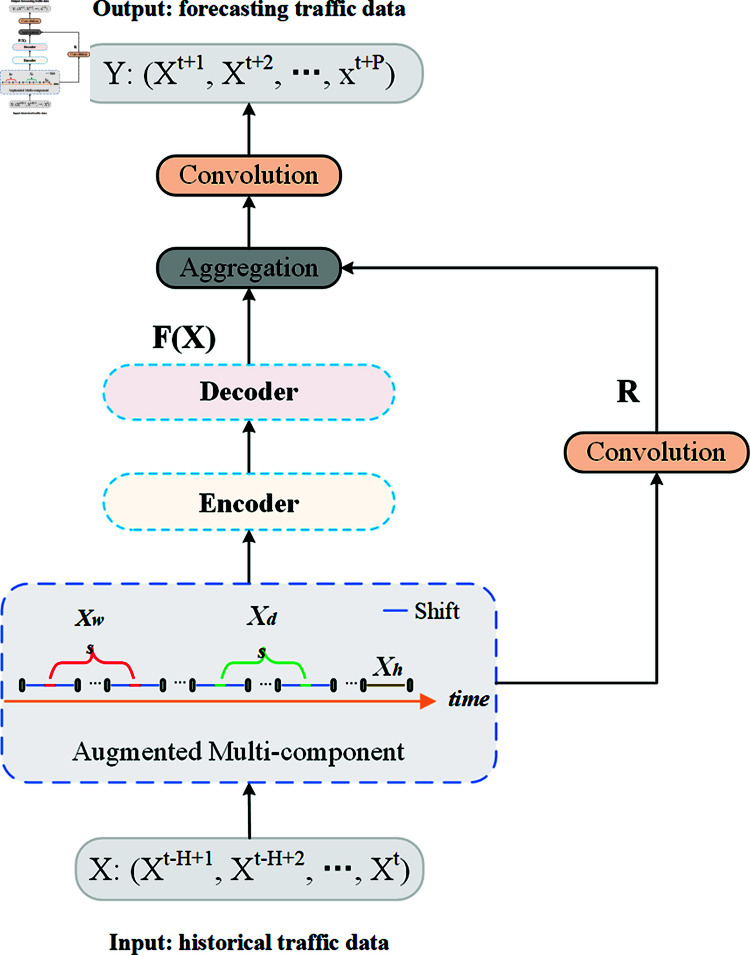
Schematic diagram of the ESGCN-EDM model structure.

This study proposes a general framework called ESGCN-EDM for predicting road traffic flow. As shown in Fig 2, the ESGCN-EDM model is built upon an encoder-decoder architecture and primarily consist of the following three modules: (1) an enhanced multi-component module designed to simultaneously capture periodic and period-shifted features; (2) an encoder module aimed at modeling the spatio-temporal feature correlations in traffic flow data; (3) a decoder module dedicated to performing multi-step predictions from spatio-temporal sequences. The internal structures of the encoder and decoder are presented in their respective sections.

### Enhanced multi-component periodic time-shift module

**Fig 3:**
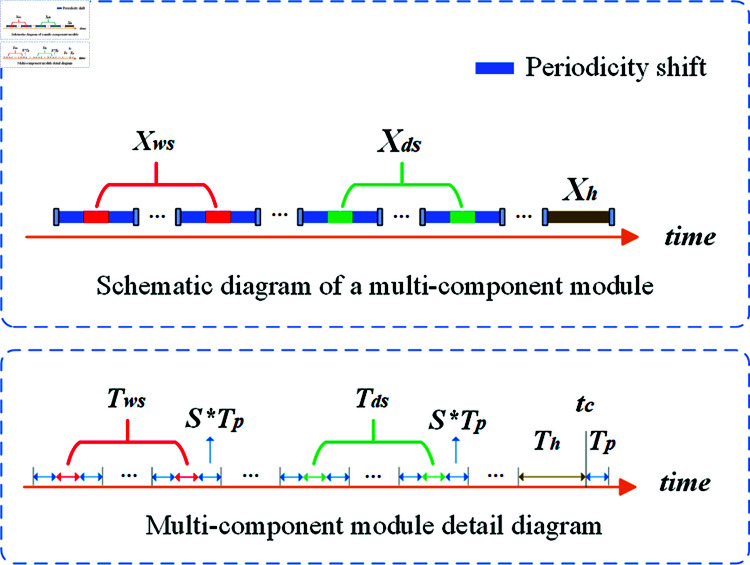
Enhanced multi-component module.

The Enhanced Multi-Component Periodic Time-shift Module is designed to capture both periodicity and time-shift characteristics in traffic flow data, thereby improving prediction accuracy [28]. Periodic time shifts refer to the dynamic temporal variations that occur within periodic patterns (e.g., daily or weekly patterns) in traffic flow data. This module captures periodic changes by analyzing traffic data at different time scales. The Enhanced Multi-Component Module consists of components derived from the recent component, the daily increment component, and the weekly increment component, with each component representing traffic flow features at different time scales. As shown in Fig 3, the upper part illustrates the schematic diagram of the enhanced multi-component module, while the lower part provides a detailed view of the enhanced multi-component module. Where, *X*_*h*_, *X*_*ds*_ and *X*_*ws*_ represent the latest time series, the daily increment component, and the weekly increment component, respectively, while *T*_*h*_, *T*_*d*_, and *T*_*w*_ denote the time steps of these three components, describing traffic flow data at different time scales. The enhanced component is defined using the time step *T*_*p*_ and the shift *s*.

The Recent Component: represents the most recent time period of the predicted sequence. Based on the continuity assumption, it suggests a strong correlation between temporally close time points.

$${𝒳}_{h}=(X^{t_{c}-T_{h}+1},X^{t_{c}-T_{h}+2},...,X^{t_{c}})\in {ℛ}^{T_{h}*N*F}$$ (3)

The Daily Increment Component: represents data from the past few days corresponding to the forecast window’s period, incorporating s to account for periodic changes.


$${𝒳}_{ds}=(X^{t_{c}-N_{d}*f-S*T_{p}+1},...,X^{t_{c}-N_{d}*f-S*T_{p}+T_{p}},$$

$$X^{t_{c}-(N_{d}-1)*f-S*T_{p}+1},...,X^{t_{c}-(N_{d}-1)*f-S*T_{p}+T_{p}},$$ (4)

$$X^{t_{c}-f-S*T_{p}+1},...,X^{t_{c}-f-S*T_{p}+T_{p}})\in {ℛ}^{T_{ds}*N*F}$$

The Weekly Increment Component: represents data from the past few weeks corresponding to the forecast window’s period, incorporating the s to capture periodicity.


$${𝒳}_{ws}=(X^{t_{c}-N_{w}*f*7-S*T_{p}+1},...,X^{t_{c}-N_{w}*f*7-S*T_{p}+T_{p}},$$

$$X^{t_{c}-(N_{w}-1)*f*7-S*T_{p}+1},...,X^{t_{c}-(N_{w}-1)*f*7-S*T_{p}+T_{p}},$$ (5)

$$X^{t_{c}-f*7-S*T_{p}+1},...,X^{t_{c}-f*7-S*T_{p}+T_{p}})\in {ℛ}^{T_{ws}*N*F}$$

where *N* is the number of nodes in the road network, *F* is the number of dimensions of each node. At any given time *t*, *X*_*t*_ denotes the traffic flow. The prediction window is *T*_*p*_, with *t*_*c*_ as the current time. *T*_*h*_, *T*_*d*_, and *T*_*w*_ correspond to the time steps for the above components, describing traffic flow data across different time scales. Specifically, $T_{h}=N_{h}\;*\;T_{p}$, where $N_{h}\in N^{+}$ represents the traffic sequence of the past *N*_*h*_ hours. $T_{d}=N_{d}\;*\;T_{P}$, where $N_{d}\in N^{+}$ signifies the traffic flow during the same time period over the past *N*_*d*_ days. $T_{w}=N_{w}\;*\;T_{p}$, where $N_{w}\in N^{+}$ indicates the traffic flow during the same time period on a specific day from the past *N*_*w*_ weeks (e.g., every Monday).

The above components collectively constitute an enhanced multi-component module, where the daily and weekly increment components account for the impact of temporal shift on traffic flow and address periodic and time-variant characteristics in traffic predictive. Let $T=T_{h}\;+\;T_{d}\;+\;T_{w}$ denote the length of the enhanced module, and the input data $X_{am}=(X_{h},X_{d},X_{w})\in {ℛ}^{T*N*F}$ is fed into the subsequent encoder-decoder architecture.

### Encoder for extracting spatiotemporal feature correlations

**Fig 4:**
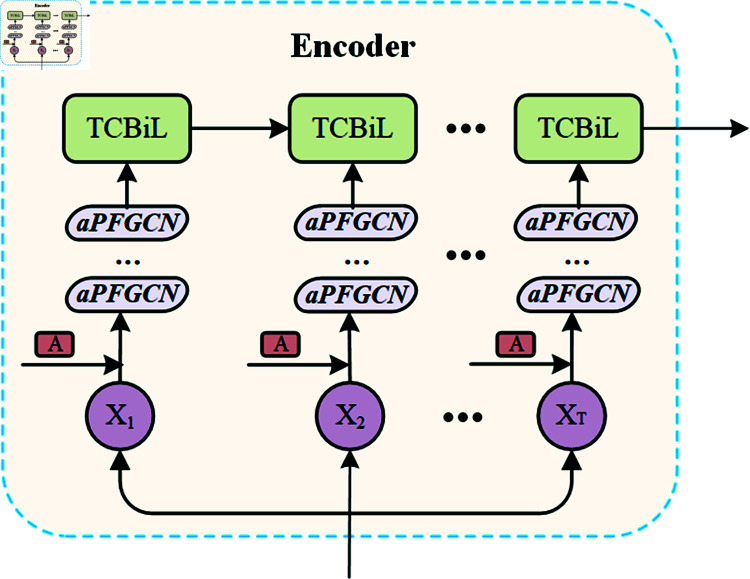
Detail of the encoder.

The encoder module aims to fully leverage spatial and temporal information within the transportation network, learning the spatio-temporal feature correlations of traffic flow data and providing the decoder with rich feature representations. As shown in Fig 4, the encoder consists of an aPFGCN module for spatial data extraction and a TCBiL module for temporal information extraction. Where, *A* represents the adjacency matrix of the road network.

#### aPFGCN for the spatial dimension.

**Fig 5:**
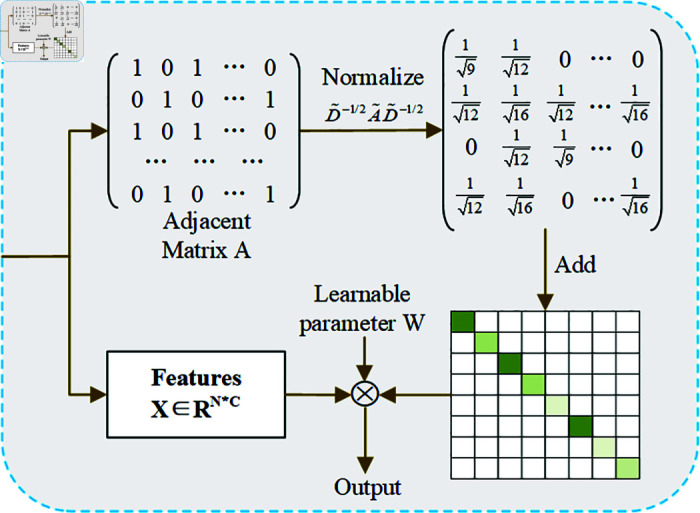
Detail of the personalized-enhanced GCN.

The traffic network is a typical graph structure, where the traffic flow of adjacent sensors is crucial for prediction. GCNs can effectively handle the graph-structured characteristics of transportation networks and capture spatial-topological relationships through simple graph convolution operations. The convolution operation in GCNs involves nodes gathering information from themselves and their neighbors, followed by updating their values. The GCN model can capture the relationships between a central node and its first-order neighbors, which can then be extended to higher-order neighbors. This capability allows GCNs to better model the spatial propagation and influence of traffic flow [38]. However, the continuous shifting of filters may slightly interfere with the overall prediction results and blur data peaks [39]. Therefore, as shown in Fig 5, this study introduces a learnable diagonal matrix Λ to personalize the values of the elements on the diagonal of the adjacency matrix.

The essence of this matrix is to achieve personalization by increasing or decreasing the diagonal element values of each node, thereby optimizing influence and ensuring the adaptability and scalability of the model. This allows the influence coefficients of each node to be dynamically adjusted to meet their unique roles within the network.

$$H^{\left(l+1\right)}=\sigma [({\tilde{D}}^{-1/2}\tilde{A}{\tilde{D}}^{-1/2}+\Lambda )H^{\left(l\right)}W^{\left(l\right)}]$$ (6)

Where $\Lambda \in {ℛ}^{N*N}$ is a learnable diagonal matrix that contains personalized weights for self-node enhancement.

During the training process, unlike the existing graph convolution operation shown in Eq (1), the model learns and obtains an enhanced matrix as shown in Eq (6), which strengthens the weights of different nodes at various levels. After obtaining the representations at different layers, the representation from the final layer is used as the ultimate representation of the Personalized-enhanced Graph Convolutional Network(PGCN). This is denoted as $X^{\prime }\in {ℛ}^{N*T*d_{G}}$, where *d*_*G*_ is the feature dimension in $X^{\prime }$.

Traffic flow data may contain various types of noise, such as data anomalies caused by sensor malfunctions. To enhance the extraction of valuable implicit features from the noise introduced by PGCN, this study first employs Fourier transform to decompose node features. The Fourier transform converts node features from the time domain to the frequency domain, where processing the features helps smooth out short-term fluctuations and random noise in the data. This enables the model to focus more on capturing long-term trends and periodic changes in traffic flow. By considering both the global structure and local connectivity of the graph, noise components become easier to identify and remove in the frequency domain, thereby improving the stability and accuracy of predictions. Additionally, this approach effectively reduces the dimensionality of the features, lowering the complexity of the model. This helps avoid overfitting, allowing the model to achieve better generalization capabilities even with limited training data, ultimately enhancing the accuracy of traffic flow prediction [40]. Finally, the inverse Fourier transform is used to remap the fused features back to the time domain, resulting in the final node feature representation. This forms the Personalized-enhanced Fusion Graph Convolutional Network (PFGCN) model, which obtains more valuable implicit feature information and resists the impact of noisy data.

**Fig 6:**
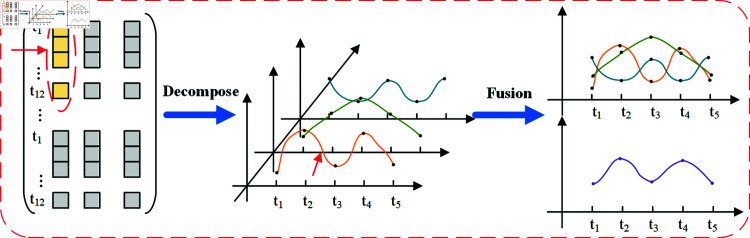
Detail of the Fourier transform.

As shown in Fig 6, the left side of the figure displays the output matrix $X^{\prime }$ generated by the PGCN model, which encapsulates various implicit features. By applying the Fourier transform, the implicit features within the matrix are systematically decomposed and captured. Subsequently, a weight matrix *W*_*Fourier*_ is used to adjust the weight of each extracted feature. By employing the inverse Fourier transform, the implicit features on the right side of Fig 6 are integrated to generate refined node features, ensuring that it can emphasize critical implicit features within a rich feature representation.

$$X_{Fourier}^{\prime }=Fus(Dec(X^{\prime })⊙X_{Fourier})$$ (7)

Where $X_{Fourier}^{\prime }\in {ℛ}^{N*T*d_{G}}$ is the output of the Fourier transform module. The *Dec*() decomposes $X^{\prime }$ into the frequency domain, selectively discarding irrelevant features. The *Fus*() reintroduces the remaining features into the time domain.

In order to maximize the topological properties of the network, a two-layer PFGCN with shared weights is employed to efficiently capture the spatial features of the traffic network in each time slice. The two-layer PFGCN model can be expressed as follows:

$$X_{Double}^{\prime }=Fus(Dec(Fus(Dec(X^{\prime })⊙X_{Fourier}))⊙X_{Fourier})$$ (8)

-1Traditional GCNs typically aggregate neighbor node features using equal weights, which may dilute the features of some important neighbor nodes while giving undue prominence to less significant features. To enhance the model’s ability to capture complex inter-node relationships and spatial structural information, the PFGCN is combined with the multi-head attention mechanism [41] to form the aPFGCN module. The multi-head attention layer computes attention weights between nodes through multiple attention heads, then aggregates these weights through weighted summation to produce the final attention-weighted feature representation. By leveraging the multi-head attention mechanism, it can better capture these complex spatio-temporal relationships, improving its ability to model spatial structural information and understand and predict traffic flow patterns. At each time step, two rounds of graph convolution and Fourier transform are performed to generate intermediate features, which are then processed through the multi-head attention layer to produce the final output features.

$$\hat{Y}=Cov(LayerNorm(MultiHead({\hat{X}}_{Double}^{\prime })+{\hat{X}}_{Double}^{\prime }))$$ (9)

The pseudo-code for the aPFGCN module is as follows:

**Algorithm 1 Attention-based personalized-enhanced fusion graph convolution network (aPFGCN).**

1: **Input:**

2:   • Adjacency matrix $A\in {\mathbb{R}}^{N\times N}$

3:   • Node feature matrix $X\in {\mathbb{R}}^{N\times D}$

4:   • Learnable diagonal matrix $\Lambda \in {\mathbb{R}}^{N\times N}$

5:   • Fourier transform weight matrix W_Fourier

6:   • Attention mechanism parameters

7: **Output:**

8:   • Node feature representation after attention fusion: aPFGCN_output = $F\in {\mathbb{R}}^{N\times D^{\prime }}$

9: **Process:**

10: Initialize adjacency matrix *A* and node feature matrix *X*.

11: Compute enhanced adjacency matrix: A_enhanced=A+Λ.

12: Perform graph convolution:

13:   a. Calculate degree matrix *D*.

14:   b. Normalize adjacency matrix: $A\_norm=D^{-1/2}*A\_enhanced*D^{-1/2}$.

15:   c. Graph convolution result: $H=\sigma (A\_norm*X*W^{\left(1\right)})$.

16: Apply Fourier transform to *H*:

17:   a. Transform *H* to frequency domain: H_freq=FFT(H).

18:   b. Feature decomposition and weight adjustment: H_filtered=H_freq*W_Fourier.

19:   c. Inverse Fourier transform back to time domain: H_time=IFFT(H_filtered).

20: Introduce attention mechanism:

21:   a. Calculate attention scores: attention_scores=Attention(H_time).

22:   b. Weighted sum of node features: F=∑(attention_scores*H_time).

23: Return fused node feature representation aPFGCN_output=F.

#### TCBiL for the temporal dimension.

**Fig 7:**
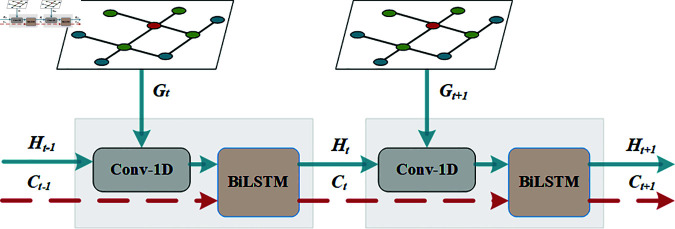
The architecture of the TCBiL.

Traffic flow data forms a three-dimensional input consisting of nodes, sequences, and features. In the previous section, the aPFGCN is used to capture spatial correlations among all sensors along the node dimension. This section leverages the TCBiL module to extract temporal correlations in traffic flow sequences. The design of the TCBiL module is partially inspired by ConvLSTM [42], and the TCBiL framework is illustrated in Fig 7. The TCBiL module integrates 1D convolution with BiLSTM to form a unified temporal feature extraction module. The 1D convolution is employed to extract local features within the time series for each sensor, while the BiLSTM is used to capture long-term dependencies in the time series. Traditional LSTMs can only gather information from past time points, whereas the bidirectional structure of BiLSTM enables the capture of information from both past and future time points, enhancing the extraction of temporal features. This bidirectional structure allows the TCBiL module to simultaneously access information from both past and future time points, improving the model’s ability to model temporal dynamics. As a result, it can not only capture the influence of historical data on the present and future but also consider the potential impact of future data on the current state, thereby enhancing its temporal modeling capabilities. Since standalone BiLSTM may not fully capture local features and short-term fluctuations when processing time series data, the TCBiL module further enhances the utilization of bidirectional information by incorporating 1D convolution, enabling the model to more accurately predict trends in time series. In this way, it can simultaneously capture short-term fluctuations and long-term trends in the time series, allowing it to more effectively extract temporal features from traffic flow data.

To ensure the model simultaneously captures spatio-temporal features, the spatial features *G*_*t*_ of the traffic flow sequence at each time step are used as input to the TCBiL module. The TCBiL first integrates the spatial features of each sensor using 1D convolution based on the previous hidden state *H*_*t*−1_ and *G*_*t*_, and then transmits this information along with the cell memory state *C*_*t*−1_ to the BiLSTM to learn temporal features. The BiLSTM consists of a forward LSTM and a backward LSTM. The forward LSTM propagates information from the beginning to the end of the time series, while the backward LSTM propagates information from the end to the beginning. The BiLSTM learns and memorizes long-term dependencies in the time series through its internal gating mechanisms (input gate, forget gate, and output gate), enabling comprehensive modeling of the time series. This bidirectional information propagation allows the model to more thoroughly capture complex features in the time series. The specific computational steps of the BiLSTM are as follows:

Forward LSTM:

$$\left\{ \begin{array}{c} I_{t}=\sigma (W_{gi}*G_{t}+W_{hi}*H_{t-1}+W_{ci}⊙C_{t-1}),\\ F_{t}=\sigma (W_{gf}*G_{t}+W_{hf}*H_{t-1}+W_{cf}⊙C_{t-1}),\\ C_{t}=F_{t}⊙C_{t-1}+I_{t}⊙tanh(W_{gc}*G_{t}+W_{hc}*H_{t-1}),\\ O_{t}=\sigma (W_{go}*G_{t}+W_{ho}*H_{t-1}+W_{co}⊙C_{t}),\\ H_{t}=O_{t}⊙tanh(C_{t}) \end{array}\right.$$ (10)

Backward LSTM:

$$\left\{ \begin{array}{c} I_{t}^{\prime }=\sigma (W_{gi}^{\prime }*G_{t}+W_{hi}^{\prime }*H_{t-1}^{\prime }+W_{ci}^{\prime }⊙C_{t-1}^{\prime }),\\ F_{t}^{\prime }=\sigma (W_{gf}^{\prime }*G_{t}+W_{hf}^{\prime }*H_{t-1}^{\prime }+W_{cf}^{\prime }⊙C_{t-1}^{\prime }),\\ C_{t}^{\prime }=F_{t}^{\prime }⊙C_{t-1}^{\prime }+I_{t}^{\prime }⊙tanh(W_{gc}^{\prime }*G_{t}+W_{hc}^{\prime }*H_{t-1}^{\prime }),\\ O_{t}^{\prime }=\sigma (W_{go}^{\prime }*G_{t}+W_{ho}^{\prime }*H_{t-1}^{\prime }+W_{co}^{\prime }⊙C_{t}^{\prime }),\\ H_{t}^{\prime }=O_{t}^{\prime }⊙tanh(C_{t}^{\prime }) \end{array}\right.$$ (11)

where *I*_*t*_, ${I^{\prime }}_{t}$, *F*_*t*_, ${F^{\prime }}_{t}$, *C*_*t*_, ${C^{\prime }}_{t}$, *O*_*t*_, ${O^{\prime }}_{t}$, *H*_*t*_, ${H^{\prime }}_{t}$ are the forward and backward input gates, forgetting gates, cell states, output gates, and hidden states, respectively. At time step *t*, *G*_*t*_ is the vector representation of the input sequence. *W* is the parameter of the model, σ is the sigmoid function, ⨀ denotes the Hadamard product.

Before processing the first input, all states of the BiLSTM are initialized to zero. The forward LSTM uses *I*_*t*_, *F*_*t*_, and *H*_*t*−1_ to update *C*_*t*_, and *O*_*t*_ to update the current *H*_*t*_. Finally, a similar process is performed using the backward LSTM.

During the training phase, zero-padding is applied to the hidden states before performing the convolution operation. The kernel size and padding size of the 1D convolution are set to 3 and 1, respectively. To ensure that spatial features can simultaneously learn spatio-temporal features, the spatial features *G*_*t*_ at each time step of the traffic flow sequence are input to the TCBiL, aiding the model in learning spatio-temporal correlations. Let *t* denote the time step; the proposed TCBiL is computed as follows:

$$H_{t},C_{t}=TCBiL(G_{t};H_{t-1};C_{t-1})$$ (12)

Where t∈{1,...,T}.

The integration of BiLSTM technology in TCBiL allows for a more comprehensive extraction of sequence information, which improves the model’s predictive performance. The pseudo-code for the TCBiL module is as follows:

**Algorithm 2 Temporal convolutional bidirectional long short-term memory (TCBiL).**

1: **Input:**

2:   • Time series data $X\in {\mathbb{R}}^{T\times N\times F}$

3:   • Number of hidden units hidden_size

4:   • Convolution kernel size kernel_size

5:   • Padding size padding_size

6: **Output:**

7:   • Temporal feature extraction result $H\in {\mathbb{R}}^{T\times N\times hidden\_size}$

8: **Process:**

9: Initialize the hidden states and cell memory states of the forward and backward LSTMs to zero.

10: **for** each time step *t* from 1 to *T* **do**

11:   a. Extract the input data of the current time step: $x_{t}=X[t]$

12:   b. Use 1-D convolution to extract local features:

13:     Convolution operation: $conv\_out=Conv1D(x_{t},kernel\_size,padding\_size)$

14:   c. Update the forward LSTM:

15:      Input gate: $i_{t}=\sigma (W_{i}*[conv\_out;h_{t-1}^{f}]+b_{i})$

16:      Forget gate: $f_{t}=\sigma (W_{f}*[conv\_out;h_{t-1}^{f}]+b_{f})$

17:      Cell state: $C_{t}^{f}=f_{t}*C_{t-1}^{f}+i_{t}*\tanh(W_{C}*[conv\_out;h_{t-1}^{f}]+b_{C})$

18:      Output gate: $o_{t}=\sigma (W_{o}*[conv\_out;h_{t-1}^{f}]+b_{o})$

19:      Hidden state: $h_{t}^{f}=o_{t}*\tanh(C_{t}^{f})$

20:   d. Update the backward LSTM:

21:      Input gate: $i_{t}^{\prime }=\sigma (W_{i^{\prime }}*[conv\_out;h_{t+1}^{b}]+b_{i^{\prime }})$

22:      Forget gate: $f_{t}^{\prime }=\sigma (W_{f^{\prime }}*[conv\_out;h_{t+1}^{b}]+b_{f^{\prime }})$

23:      Cell state: $C_{t}^{b}=f_{t}^{\prime }*C_{t+1}^{b}+i_{t}^{\prime }*\tanh(W_{C^{\prime }}*[conv\_out;h_{t+1}^{b}]$ $+b_{C^{\prime }})$

24:      Output gate: $o_{t}^{\prime }=\sigma (W_{o^{\prime }}*[conv\_out;h_{t+1}^{b}]+b_{o^{\prime }})$

25:      Hidden state: $h_{t}^{b}=o_{t}^{\prime }*\tanh(C_{t}^{b})$

26:   e. Concatenate the hidden states of the forward and backward LSTMs: $h_{t}=[h_{t}^{f};h_{t}^{b}]$

27:   f. Use *h*_*t*_ as the output of the current time step

28: **end for**

29: Return the temporal feature extraction result *H*

### Decoder for multi-step prediction

**Fig 8:**
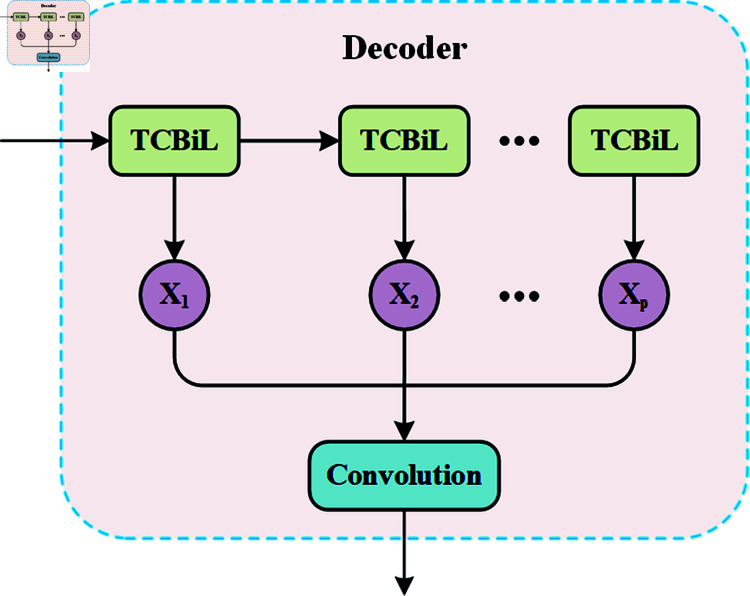
Detail of the decoder.

The decoder module aims to generate an accurate traffic flow forecasting sequence through multi-step predictions using the spatiotemporal features extracted by the encoder. As shown in Fig 8, the decoder part consists mainly of two main parts: TCBiL module and CNN. The role of TCBiL is to unfold *H*_*T*_ and *C*_*T*_, and CNN is used to convert the multi-step prediction into a high-dimensional feature representation. By combining TCBiL and CNN, the decoder module effectively generates high-dimensional feature representations from spatiotemporal sequences and facilitates multi-step forecasting.

Since TCBiL has no input sequence, an all-zero array matching the dimensions of the hidden state *H*_*T*_ is initialized as a simplified input. Specifically, at each time step, the hidden and cell memory states from the previous moment, together with the all-zero array, are used to predict the next moment, ensuring that predictions at each time step are related to the previous state. Assuming the prediction window size to be *P*, the formulation of the decoder is as follows:

$$X_{t-1}=TCBiL(0;H_{t};C_{t})$$ (13)

Where t∈{T,...,T+P−1} and 0 represents all-zero arrays. Then, all outputs are concatenated into the sequence $\{X_{T},...,X_{T+P}\}\in {ℛ}^{P*N*H}$, and a convolutional operation is conducted to transform the multi-step predictions into a high-dimensional representation.

### Fusion module

The high-dimensional representations obtained from the aforementioned enhanced multi-component module and the encoder-decoder structure are passed to a fusion module composed of residual connections and CNNs to generate the prediction results. The fusion module integrates the residual information *R* from the enhanced multi-component module with the high-dimensional representation *F*(*X*) from the decoder using convolutional residual connections, thereby accelerating model training and mitigating overfitting issues. Ultimately, a CNN is employed to ensure that the prediction results $Y\in {ℛ}^{P*N*F}$ have the same dimensions and shape as the expected output.

Y=Conv(R+F(X)) (14)

Where *Y* is the final prediction outcome, containing multi-step forecasts from *t* + 1 to *t* + *p*. *Conv* denotes a convolution with 1*1 kernel size. + signifies a residual connection that combines the residual information *R* with the decoder’s high-dimensional feature representation *F*(*X*).

The fusion module further enhances the model’s performance by leveraging residual connections to integrate the residual information from the enhanced multi-component module with the high-dimensional representation from the decoder. Additionally, the CNN adjusts the dimensions and shape of the final prediction results to ensure consistency with the expected output, improving training efficiency and effectively reducing the risk of overfitting.

## Experimental results and analysis

To validate the performance of the ESGCN-EDM model, this study conducts experimental comparisons on two real-world public transportation datasets and performs ablation experiments on different modules within the ESGCN-EDM model to verify the effectiveness of each module. To assess the rationality of the model parameters, a sensitivity analysis experiment is conducted on the time window parameters used in the experiments.

### Experimental dataset

Due to the limitation of experimental resources (e.g., computational resources, time, etc.), the datasets used in the experiments are the public transportation datasets PeMSD4 and PeMSD8 collected by PeMS in California, USA. These datasets cover highway traffic conditions in all major cities in California, provided by over 39,000 independent sensors. The sensors cover the highway systems of major metropolitan areas in California, with data samples recorded every 5 minutes, including sensor observations and geographic information. This ensures that the results are reproducible and transparent. The two datasets have different geographic characteristics and traffic flow patterns, enabling the validation of the model’s generalization capabilities across diverse traffic environments. With limited experimental resources, the use of the two datasets allows for adequate experimentation within reasonable computational resources and timeframe, while ensuring the reliability and representativeness of the experimental results.

The study uses the benchmark datasets PeMSD4 and PeMSD8, as published by Guo *et al*. [21], which have eliminated redundant sensors spaced less than 3.5 miles apart and have linearly interpolated missing values. Table 1 lists the specifics of the dataset: (1) PeMSD4 collects traffic flow data of the San Francisco Bay Area from January 1, 2018 to February 28, 2018 involving 307 sensors. In this paper, the first 50 days of PeMSD4 are selected to construct the training and validation sets, and the remaining 9 days are used as the test set. (2) PeMSD8 contains two months of traffic flow data for the San Bernardino area from July 1, 2016 to August 31, 2016, monitored by 170 sensors. Similarly, the first 50 days of PeMSD8 are chosen for the training and validation sets, and the remaining 12 days of data are used as the test set. In addition, the maximum value *Max*(*X*) in the datasets is calculated, and the entire dataset is preprocessed by normalization using X=X/Max(X).

**Table 1:** Dataset description.

Datasets	Nodes	Edges	Interval	Time Range		Time Steps
PeMSD4	307	340	5min	2018/1/1	2018/2/28	16992
PeMSD8	170	295	5min	2016/7/1	2016/8/31	17856

### Model parameters

All models are compiled and tested experimentally based on Pytorch. Experiments are conducted on a server with GPU NVIDIA GeForce RTX 2080 Ti and CPU 2.40GHz Intel(R) Xeon(R). The parameter configuration of the model is divided into three main parts:

(1) Enhanced Multi-Component: This study focuses on predicting traffic flow for the next hour, with *T*_*p*_ set to 12. The model parameters for *T*_*p*_ = 6 and *T*_*p*_ = 3 are consistent with those for *T*_*p*_ = 12 to enhance training efficiency. For both datasets, the parameters for the three components are *T*_*h*_ = 24, *T*_*d*_ = 12, and *T*_*w*_ = 12, and a periodic offset of *s* is applied. The length of the enhanced multi-component sequence is *T*_*D*_ = 96.

(2) Network Structure: The encoder uses a two-layer aPFGCN with a number of convolution filters of 128 and 64, respectively. TCBiL’s convolutional filters match the number of sensors, with each filter having 64 hidden units. In the decoder, TCBiL also features 64 hidden units, and the output sequence is *T*_*p*_ in length. The convolutional filter of the CNN within the decoder is the same as the sequence length *T*_*D*_. In the fusion module, convolutional filters of size *T*_*p*_ are utilized.

(3) Training Hyperparameters: The model utilizes the Adam optimizer [43], configured with a learning rate of 0.004, a weight decay of 0.003, a batch size of 16, and a dropout rate of 0.6 [44]. The deviation between predicted and true values is evaluated using the Mask Huber function [45]:

$$loss(Y,Y^{\prime })=\left\{ \begin{array}{cc} {\frac{1}{2}(Y-Y^{\prime })}^{2}, & |Y-Y^{\prime }|⩽\delta \\ \delta |Y-Y^{\prime }|-\frac{1}{2}\delta^{2}, & otherwise \end{array}\right.$$ (15)

Where *Y* is the true values and $Y^{\prime }$ is the predicted values; δ is a threshold for sensitivity to error, which took the value of 3.0 in the experiment.

### Evaluation metrics

To evaluate the performance of the model, the experiment employs three commonly used regression evaluation metrics: Mean Absolute Error (MAE), Root Mean Square Error (RMSE), and Symmetric Mean Absolute Percentage Error (SMAPE).

MAE reflects the average deviation between the predicted values and the actual values, and its results are easy to be understood and compared. Since traffic flow data usually have a certain degree of volatility, MAE can provide a stable error metric, which is insensitive to the outliers, and shows how much the model deviates from the real flow in general. the extent to which the model deviates from the true flow in general, and is suitable for assessing the performance of the model in most situations [46].

RMSE is more sensitive to larger prediction errors and is suitable for assessing model performance in extreme cases. In addition, the relationship between RMSE and variance allows it to reflect the degree of dispersion of forecast errors, helping us to assess the stability and consistency of the model [47].

SMAPE reflects the average magnitude of relative error between predicted and actual values. Its results are presented as a percentage, typically ranging from 0% to 100%, making it easy to interpret and compare. SMAPE provides a fairer evaluation of a model’s predictive performance across data of varying scales. By assigning equal weight to cases where predictions are either higher or lower than the actual values, SMAPE addresses the asymmetry issue inherent in traditional MAPE. This makes SMAPE a more balanced and comprehensive metric for assessing the overall performance of models [48].

The combined use of these three metrics provides a comprehensive evaluation of the model’s predictive performance. By comparing the performance of different models across these metrics, a more accurate judgment of model superiority can be made, offering valuable insights for research and applications in traffic flow prediction. The calculation methods for MAE, RMSE, and SMAPE are as follows:

$$MAE=\frac{1}{n}\sum_{i=1}^{n}|Y_{i}-Y_{i}^{\prime }|$$ (16)

$$RMSE=\sqrt{\frac{1}{n}\sum_{i=1}^{n}{\left(Y_{i}-Y_{i}^{\prime }\right)}^{2}}$$ (17)

$$SMAPE=\frac{100\%}{n}\sum_{i=1}^{n}\frac{\left|Y_{i}-Y_{i}^{\prime }\right|}{\left(\left|Y_{i}\right|+\left|Y_{i}^{\prime }\right|\right)/2}$$ (18)

Where *n* is the number of all predictions.

### Baseline models

In this paper, the proposed ESGCN-EDM model is compared with the following baseline model:

**LSTM**: A specialized RNN model for time series prediction, which addresses the issues of vanishing and exploding gradients in traditional RNNs when processing long sequences by introducing input gates, forget gates, and output gates. This enables LSTM to effectively capture long-term dependencies in time series data. Since its introduction in 1997, LSTM has become a classic model in the field of time series prediction and has been widely applied in various domains such as stock price forecasting, weather prediction, and speech recognition. Its advantages in handling time series data have been widely recognized by both academia and industry. In this study, the historical traffic flow time window is set to *T*_*h*_ = 12, and the number of hidden layers is set to *h* = 64.

**GRU** [49]: An enhanced RNN model optimized for time series forecasting, which simplifies the three gating mechanisms in LSTM to two: the update gate and the reset gate. This reduction in model parameters enhances training efficiency. The GRU’s effectiveness and accuracy in processing time series data have been acknowledged by the academic community, and it has demonstrated excellent performance in numerous practical applications. The parameters are configured as *T*_*h*_ = 12 and *h* = 64.

**MCSTGCN** [50]: A multi-component network for traffic flow prediction, which integrates GCN and time series models to simultaneously capture spatial and temporal features in traffic networks. This approach enables the model to better capture the periodicity and time-varying characteristics of traffic flow. The parameters are set as *T*_*h*_ = 36, *T*_*d*_ = 12, and *T*_*w*_ = 12.

**ASTGCN** [15]: A traffic flow prediction model that integrates attention mechanisms with graph convolutional networks. By incorporating attention mechanisms, it can more flexibly capture spatiotemporal correlations in traffic networks. The introduction of attention mechanisms allows the model to better adapt to the variability in traffic flow across different regions. The parameters are set as *T*_*h*_ = 24, *T*_*d*_ = 12, and *T*_*w*_ = 24.

**AMRGCN** [28]:An enhanced multi-component recurrent graph convolutional network model designed for traffic flow prediction. By integrating graph convolutional networks with recurrent neural networks, it effectively captures spatiotemporal features in traffic networks. The model’s multi-component design and enhanced recurrent architecture enable it to better capture the periodicity and time-shift characteristics of traffic flow. The parameters are configured as *T*_*h*_ = 24, *T*_*d*_ = 12, and *T*_*w*_ = 12.

### Experimental results and discussion

Overall, the ESGCN-EDM model achieves the best performance in PeMSD4 and PeMSD8 compared to existing good deep learning methods. The effectiveness of the aPFGCN and TCBiL modules are evidenced by: a) The aPFGCN method’s superiority over the standard GCN method in capturing spatial correlations within traffic flow; b) The TCBiL module’s ability to effectively learn spatiotemporal correlations, proving superior to other variants.The experimental results demonstrate that the proposed ESGCN-EDM model achieves superior performance on both the PeMSD4 and PeMSD8 datasets compared to existing state-of-the-art deep learning methods. Through the evaluation of the aPFGCN and TCBiL modules, the following aspects are validated: a) the aPFGCN method outperforms standard GCN methods in capturing spatial correlations in traffic flow; b) the TCBiL module effectively learns temporal dependencies, proving its superiority over other variants. This section provides a detailed discussion of the key findings and their implications.

#### Discussion of baseline comparison.

Table 2 presents the MAE, RMSE, and SMAPE results of the ESGCN-EDM model and baseline models for traffic flow prediction at different time intervals (15min, 30min, and 60min) on the PeMSD4 and PeMSD8 datasets. Lower values of MAE, RMSE, and SMAPE indicate higher prediction accuracy of the model. MAE reflects the average error of the model under most conditions, while RMSE places greater emphasis on the model’s performance during peak hours or under exceptional circumstances. SMAPE is particularly suitable for traffic flow data characterized by significant fluctuations and varying magnitudes. The bolded data in the table represents the best-performing results.

**Table 2:** Performance comparison of different models on PeMSD4 and PeMSD8 at different forecasting interval.

Datasets	Method	15 min			30 min			1h		
		RMSE	MAE	SMAPE	RMSE	MAE	SMAPE	RMSE	MA E	SMAPE
PeMSD8	LSTM	25.29	17.52	11.11%	27.70	19.24	12.02%	31.56	22.00	13.74%
	GRU	25.24	17.40	11.07%	27.72	19.32	12.32%	31.31	21.80	13.86%
	MCSTGCN	22.59	14.75	9.99%	23.25	15.64	10.01%	24.11	16.07	11.08%
	ASTGCN	22.81	15.05	9.91%	24.42	15.92	10.33%	24.81	16.31	10.86%
	AMRGCN	21.28	13.88	8.46%	22.03	14.78	9.38%	22.70	15.02	10.70%
	**ESGCN-EDM**	**21.07**	**12.99**	**7.66%**	**21.58**	**13.74**	**8.21%**	**22.22**	**13.84**	**8.89%**
PeMSD4	LSTM	33.71	22.21	16.17%	35.29	23.18	16.37%	38.82	25.95	18.00%
	GRU	33.81	22.24	16.23%	35.43	23.49	16.72%	38.66	25.91	18.03%
	MCSTGCN	29.81	18.49	12.89%	30.79	19.93	13.54%	31.21	20.80	14.06%
	ASTGCN	29.33	18.50	12.41%	30.70	19.92	13.14%	31.42	21.00	14.31%
	AMRGCN	27.80	18.00	11.73%	29.13	18.66	12.14%	29.48	19.42	13.49%
	**ESGCN-EDM**	**27.49**	**17.67**	**10.24%**	**28.59**	**18.14**	**11.33%**	**29.17**	**19.07**	**13.09%**

From the analysis of Table 2, it is evident that the traffic flow prediction error increases as the prediction horizon extends. In the tasks of 15min, 30min, and 1h traffic flow prediction, the proposed model outperforms other baseline models on both the PeMSD4 and PeMSD8 datasets. The MAE results demonstrate that the ESGCN-EDM model provides more accurate predictions under most conditions, while the RMSE results indicate that the ESGCN-EDM model also excels during peak hours and exceptional scenarios. These findings fully illustrate the accuracy and effectiveness of the ESGCN-EDM model.

RNN-based models (LSTM and GRU) focus only on temporal features, neglecting the spatiotemporal correlations inherent in traffic flow, which limits their performance. In contrast, GCN-based models (MCSTGCN, ASTGCN, and AMRGCN) leverage graph structures to represent the non-Euclidean nature of traffic networks. This enables the models to more effectively capture the spatiotemporal relationships between traffic nodes, resulting in generally superior performance compared to RNN-based models.

Among GCN-based models, MCSTGCN and ASTGCN are capable of capturing daily and weekly periodic patterns, but their performance is less prominent compared to AMRGCN, which incorporates time-shift periodicity. This highlights the importance of simultaneously considering periodicity and time-shift periodicity in traffic flow prediction. However, while AMRGCN utilizes GCN and conventional temporal modules to process spatial and temporal features, the proposed model in this study not only employs enhanced multi-component features to capture periodic shifts but also integrates TCBiL and aPFGCN to learn spatiotemporal correlations. As a result, AMRGCN exhibits limited effectiveness in handling spatiotemporal correlations compared to the proposed model. In the 1-hour long-term prediction task, under the same model parameters, the ESGCN-EDM model achieves a reduction of 7.9% in MAE, 2.1% in RMSE and 16.9% in SMAPE on the PeMSD8 dataset, and a reduction of 1.8% in MAE, 1.1% in RMSE and 3.0% in SMAPE on the PeMSD4 dataset, compared to the well-performing AMRGCN model. Based on the above comparison, the ESGCN-EDM model outperforms other comparison models at each time step. In the spatial dimension, the aPFGCN module of the ESGCN-EDM model dynamically adjusts the node influence coefficients through a learnable diagonal matrix, addressing the issue of feature dilution for key nodes in traditional GCNs. By incorporating Fourier transformations to convert features into the frequency domain, it effectively filters short-term noise and enhances the model’s ability to capture long-term trends. Additionally, the multi-head attention mechanism focuses on key spatial topological relationships (such as intersections and main roads), improving feature extraction efficiency in complex road networks. In the temporal dimension, the TCBiL module combines 1D convolution and BiLSTM: 1D convolution extracts local fluctuations (such as sudden congestion), while BiLSTM models bidirectional long-term dependencies. Based on bidirectional information fusion, the forward direction captures historical trends, and the backward direction associates with potential future impacts (such as the symmetry of morning and evening rush hours), making temporal modeling more comprehensive. The synergy of these components allows the ESGCN-EDM model to demonstrate significant advantages in capturing the spatiotemporal features of traffic flow sequences.

#### Sensitivity analysis.

**Fig 9:**
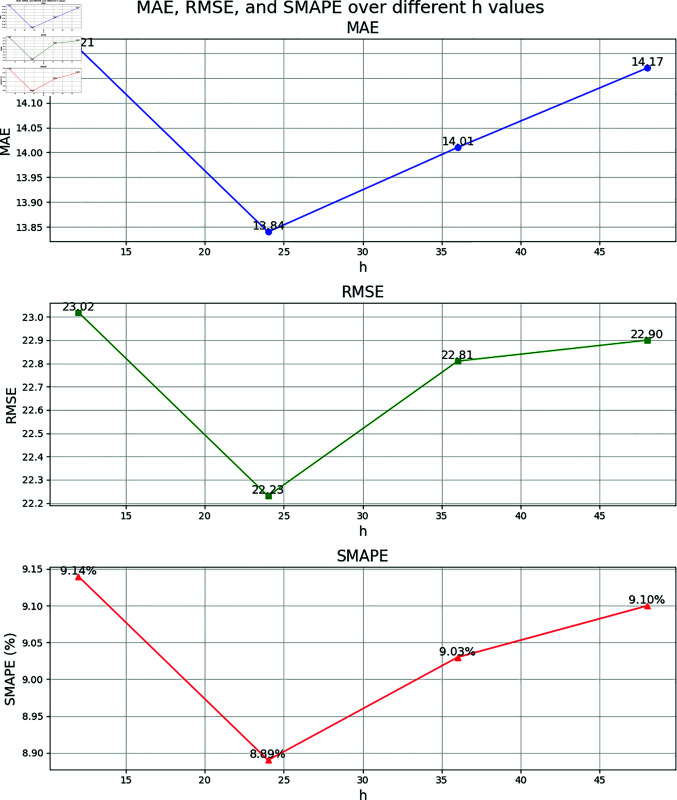
Parameter sensitivity analysis results for *T*_*h*_ (PeMSD8 dataset, 1-hour prediction).

**Fig 10:**
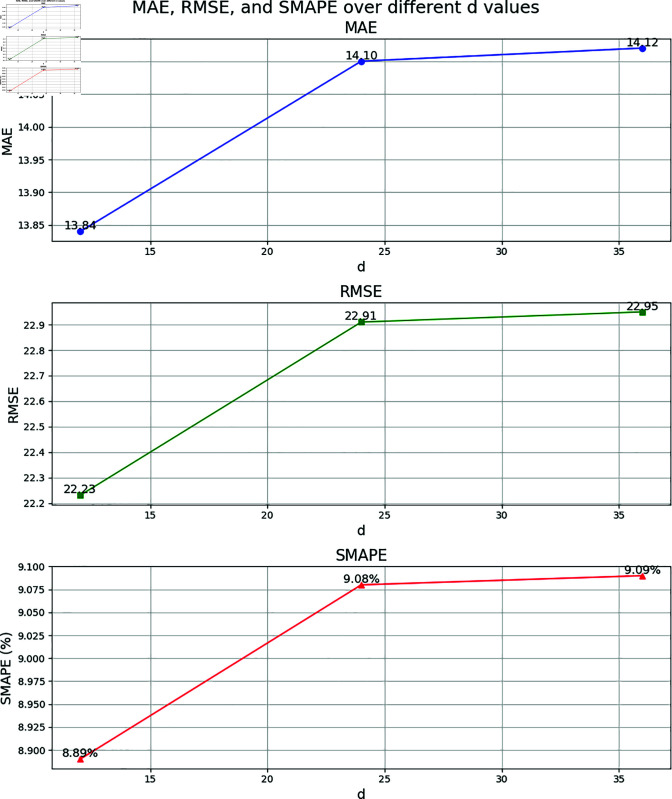
Parameter sensitivity analysis results for *T*_*d*_ (PeMSD8 dataset, 1-hour prediction).

**Fig 11:**
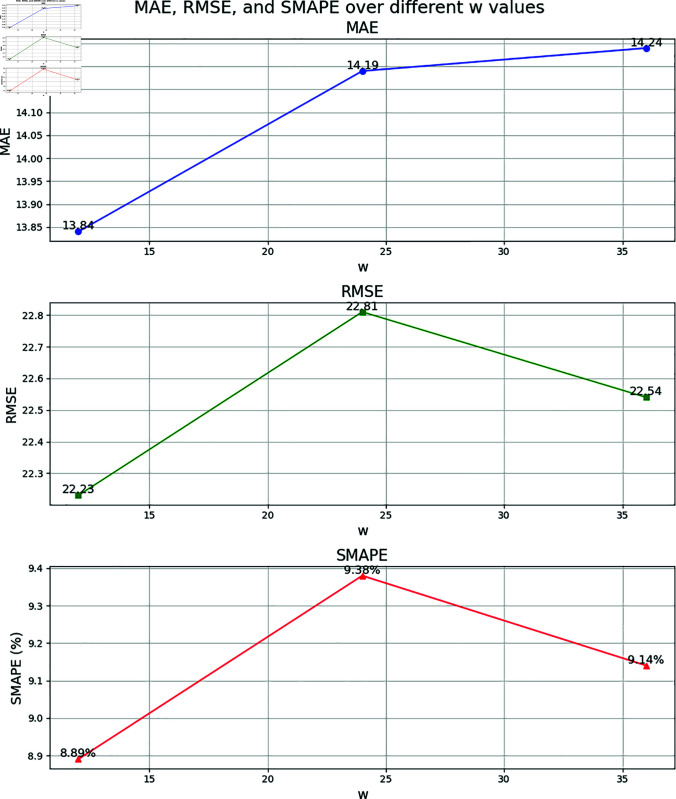
Parameter sensitivity analysis results for *T*_*w*_ (PeMSD8 dataset, 1-hour prediction).

To verify the reasonableness of the model parameters, including the time window parameters *T*_*h*_, *T*_*d*_, and *T*_*w*_, a sensitivity analysis experiment is conducted on the ESGCN-EDM model. The selection of initial parameters is based on the analysis of traffic flow data characteristics. Traffic flow typically exhibits significant short-term correlations and periodicity. Referring to classical studies in the field of traffic flow prediction (e.g., [12] [28] [50]), to capture the short-term fluctuations and immediate changes in traffic flow, the recent time window is usually set between 30 minutes to 2 hours. The daily and weekly periodic windows are intended to select data from specific time periods within a day or week to complement the periodic variations in traffic flow and balance periodicity with noise, generally ranging from 30 minutes to 2 hours. For example, since the traffic flow data is sampled every 5 minutes, *T*_*h*_=24 corresponds to the past 2 hours of traffic flow data (with each 5-minute interval representing a time step), effectively capturing short-term fluctuations. *T*_*d*_=12 and *T*_*w*_=12 represent the traffic data for the same time period over the past 1 day and 1 week, respectively, allowing the model to better capture these long-term periodic patterns. By adjusting the values of each parameter, the influence on the 1-hour traffic flow prediction performance on the PeMSD8 dataset is tested. The experiment employs a single-factor experimental design, where only one parameter is altered at a time while keeping all other parameters constant, to observe changes in model performance. To present the results of the sensitivity analysis more intuitively, the model performance under different parameter settings is displayed in the form of line charts. Figs 9, 10, and 11 show the impact of *T*_*h*_, *T*_*d*_, and *T*_*w*_ on model performance, respectively.

Sensitivity Analysis of Recent Time Window Parameter *T*_*h*_: *T*_*h*_ represents the time step for the model to capture recent traffic flow patterns. The experiment tests the performance variations when *T*_*h*_ is set to 12, 24, 36, and 48. The results show that when *T*_*h*_=24, the model achieves optimal values in MAE (13.84), RMSE (22.23), and SMAPE (8.89%). As *T*_*h*_ increases to 36 or 48, all indicators decline to varying degrees (for example, when *T*_*h*_=48, MAE increases to 14.17, and SMAPE increases to 9.10%). This indicates that excessively long recent time windows may introduce redundant information or noise, while *T*_*h*_=24 effectively balances the utilization of short-term fluctuations and historical information, capturing the traffic correlation of adjacent time steps efficiently.

Sensitivity Analysis of Daily Increment Component Parameter *T*_*d*_: *T*_*d*_ is used to model daily periodic features. The experiment sets *T*_*d*_ to 12, 24, and 36, with the results showing that when *T*_*d*_=12, the model performs best (MAE=13.84, RMSE=22.23, SMAPE=8.89%). As *T*_*d*_ increases, MAE and SMAPE increase to 14.12 and 9.09%, respectively. This suggests that increasing the historical days may introduce periodic shifts unrelated to the current prediction period, causing the model to struggle in accurately capturing daily patterns. Therefore, *T*_*d*_=12 effectively leverages single-day periodic information while avoiding overfitting issues.

Sensitivity Analysis of Weekly Increment Component Parameter *T*_*w*_: *T*_*w*_ is used to capture weekly periodic features. The experimental results show that when *T*_*w*_=12, the model achieves optimal performance (MAE=13.84, RMSE=22.23, SMAPE=8.89%). As *T*_*w*_ increases to 24 or 36, SMAPE increases to 9.38% and 9.14%, respectively. This indicates that weekly periodic features have been adequately modeled within a single-week time step, and increasing the number of weeks may lead to mismatches between the long-term trends in the historical data and the current period, reducing prediction accuracy.

Comprehensive Parameter Optimization Analysis: The experiment further validates the reasonableness of the parameter combinations. When *T*_*h*_=24, *T*_*d*_=12, and *T*_*w*_=12, the model achieves optimal performance in MAE, RMSE, and SMAPE. This suggests that the synergistic effect of the recent, daily, and weekly components effectively captures the multi-scale spatial-temporal dependencies of traffic flow. Additionally, the parameter sensitivity analysis confirms the model’s robustness to hyperparameter selection: when parameters are adjusted within a reasonable range, performance fluctuations are minimal (for example, when *T*_*h*_=36, SMAPE increases by only 0.14%), but performance significantly declines once the optimal range is exceeded (for example, when *T*_*w*_=24, SMAPE increases by 0.49%).

In conclusion, the sensitivity analysis demonstrates that the parameter settings of Th=24, Td=12, and Tw=12 in the model are reasonable and effective. These settings balance the model’s ability to capture multi-scale spatial-temporal features and computational efficiency, thereby improving prediction accuracy.

#### Ablation experiment.

To deeply explore the performance of the proposed aPFGCN and TCBiL modules, this study configures three variant models of the ESGCN-EDM on the PeMSD8 dataset with identical parameter settings for the ablation study:

a) The TCBiL component in the ESGCN-EDM model, which is responsible for extracting temporal correlations, is replaced with LSTM and TCL (TCL consists of CNN and LSTM), resulting in models named AM-LSTM-aPFGCN and AM-TCL-aPFGCN, respectively;

b) The aPFGCN module in the proposed model, which captures spatial correlations, is replaced with GCN, resulting in a model named AM-TCBiL-GCN;

c) The sampling interval for the experiment is set to 1 hour, with the goal of predicting traffic flow for the next 1 hour. The ablation study results are presented in Table 3.

**Table 3:** Comparative analysis of predictive performance of different variant models.

Method	RMSE	MAE	SMAPE	Dataset
AM-LSTM-aPFGCN	29.74	19.40	11.75%	PeMSD8
AM-TCL-aPFGCN	23.07	14.20	9.16%	
AM-TCBiL-GCN	22.53	14.28	9.41%	
ESGCN-EDM	22.22	13.84	8.89%	

Based on the results results from Table 3, the following conclusions can be drawn:

a) The AM-TCL-aPFGCN variant model performs slightly better than the AM-LSTM-aPFGCN variant. This is because the AM-LSTM-GCN variant directly concatenates the spatial features with other features into a one-dimensional when processing the spatial features from aPFGCN, which leads to the loss of spatial information. In contrast, the AM-TCL-aPFGCN variant combines one-dimensional convolution with LSTM, effectively capturing the internal features of each sensor, thus improving prediction performance.

b) The proposed model in this study outperforms the AM-TCL-aPFGCN variant. This is attributed to the limited ability of LSTM to repair and reuse data, while BiLSTM integrates bidirectional traffic flow information, enabling the reuse of historical traffic flow data, which improves the accuracy of traffic flow prediction.

c) The model proposed in this paper outperforms the AM-TCBiL-GCN variant. This is because aPFGCN integrates a personalized matrix and Fourier transform techniques, ensuring model adaptability and emphasizing more critical implicit features. Additionally, the multi-head attention layer enhances the model’s ability to capture spatial structural information, making the model’s spatial information extraction capability superior to that of a standard GCN. Therefore, ESGCN-EDM excels in handling spatiotemporal dependencies compared to the other three variant models.

## Conclusion

To address the complex spatiotemporal dependencies in traffic networks, a traffic flow prediction model based on a spatiotemporal encoder-decoder framework, ESGCN-EDM, is proposed, achieving higher accuracy in traffic flow prediction. The conclusions are as follows:

1) aPFGCN: Building upon GCN, aPFGCN incorporates a learnable diagonal matrix Λ into the adjacency matrix, allowing personalized enhancement of self-loop weights for each node. By combining Fourier transform and inverse transform, it more effectively extracts intrinsic implicit features, enhancing the model’s robustness. The integration of multi-head attention mechanisms enables the model to comprehensively understand spatial correlations in the network, strengthening its ability to capture spatial structural information.

2) TCBiL: The TCBiL module combines BiLSTM with 1D convolution to account for bidirectional traffic flow information and improve prediction accuracy, enabling simultaneous feature extraction and temporal modeling. The 1D convolution extracts short-term fluctuations and local patterns in time series, while BiLSTM captures long-term trends and periodic changes. By integrating local feature extraction from 1D convolution and long-term dependency modeling from BiLSTM, the TCBiL module effectively consolidates internal sensor features, comprehensively capturing complex characteristics in time series.

3) ESGCN-EDM Model: The encoder of the ESGCN-EDM model utilizes both aPFGCN and TCBiL to more effectively capture spatiotemporal correlations between nodes. The decoder employs TCBiL to unfold hidden states and cell memory states, combined with CNN to generate high-dimensional representations, producing accurate traffic flow prediction sequences. Experiments on the large-scale real-world dataset PeMS demonstrate that, compared to existing state-of-the-art methods, ESGCN-EDM effectively models spatiotemporal correlations in road networks and improves the accuracy of traffic flow prediction, proving its effectiveness and feasibility in traffic flow prediction tasks.

Although the ESGCN-EDM model has achieved significant results in traffic flow prediction, there are still some limitations in this study. First, the performance of the model may not be stable enough when dealing with traffic flow prediction under abnormal conditions such as extreme weather or unexpected events. Second, the training and inference process of the model is relatively complex and consumes a large amount of computational resources, which may limit its wide deployment in real-time applications. Future research will be devoted to optimizing the computational efficiency of the model and exploring its application potential in more complex scenarios.

## Supporting information

S1 File PeMSD4. Two months of traffic flow datasets for the San Francisco Bay Area, from January 1, 2018 to February 28, 2018, and includes information from 307 sensors.

S2 File PeMSD8. Traffic flow datasets from the San Bernardino area over a two-mTonth period, from July 1, 2016, to August 31, 2016, monitored by 170 sensors.
